# Advances in Magnetic Nanomaterials, Ferrofluids, and Ferrogels: From Structure to Biomedical and Engineering Applications

**DOI:** 10.3390/gels12050385

**Published:** 2026-05-01

**Authors:** Zhizheng Gao, Kun Li, Wenbo Xu, Ling Li, Wenguang Yang, Jun Li

**Affiliations:** 1 School of Electromechanical and Automotive Engineering, Yantai University, Yantai 264005, China; gaozhizheng2024@163.com (Z.G.); 17866857776@163.com (K.L.); 19937995210@163.com (W.X.); yangwenguang@ytu.edu.cn (W.Y.); 2 School of Intelligent Manufacturing, Weifang Institute of Technology, Weifang 261000, China; jessicli1988@163.com

**Keywords:** magnetic nanomaterials, ferrogels, magnetic hydrogels, sol–gel transition, soft robotics, biomedical applications

## Abstract

This review comprehensively examines magnetic nanomaterials, ferrofluids, and their integration into ferrogel systems, systematically exploring their structural characteristics, dynamic behaviors, preparation techniques, and applications across medical and engineering fields. Structural characterization reveals that particle size and dispersibility directly influence functional efficiency in fluid and gel matrices, such as SAR (specific absorption rate) values in hyperthermia applications. For ferrofluids and magnetic gels, macroscopic behaviors and microscopic mechanisms are governed by key parameters like the magnetic Bond number. Preparation encompasses green synthesis, chemical reagent synthesis, and the cross-linking of these nanoparticles into hydrogel networks. Applications span diverse areas: in medicine, these include targeted hyperthermia, pH-responsive magnetic gel drug delivery, and MRI (magnetic resonance imaging); in engineering, applications range from efficient extraction and triboelectric power generation to magnetically regulated heat transfer and soft gel robotics. The paper also discusses current challenges, including material stability and unclear micro–macro correlations in complex fluid–gel systems, outlining future research directions for multifunctional magnetic materials.

## 1. Introduction

Ferrofluid is a colloidal system formed by the stable dispersion of nanoscale magnetic particles in a carrier liquid, exhibiting both the fluidity of liquids and magnetic responsiveness [1,2]. It was originally invented for magnetic flux leakage detection of ferromagnetic components such as pipelines, railroad tracks, and storage tanks, which also represents the classic scenario where this material first achieved industrial application [3,4]. Its distinctive magnetically tunable flow field characteristics, heat transfer capabilities, and interfacial behaviors endow it with enormous application potential across fields including energy conversion, environmental governance, biomedicine, and mechanical engineering [5,6,7,8,9]. To expand their utility and structural stability, researchers increasingly encapsulate these magnetic colloidal systems within three-dimensional polymer networks to create ferrogels or magnetic hydrogels. In recent years, with the advancement of preparation technologies and the development of multi-physics field coupling theory, pivotal progress has been made in the research of ferrofluids and their cross-linked gel systems [5,10]. The controllable synthesis of magnetic nanomaterials has undergone a leap from the regulation of single magnetic properties to multifunctional surface modification [11]. The multi-field coupling behavior mechanism of ferrofluids has been expanded from the description of macroscopic phenomena to the quantitative clarification of microscopic structural evolution [12]. Ferrogel systems have achieved a breakthrough from simple magnetic composites to multi-stimuli responsive intelligent functionalization, and their application scenarios have expanded from traditional engineering fields to the frontier of precision biomedicine [13]. This field has now become one of the core directions of interdisciplinary research, covering materials science, fluid mechanics, biomedicine, and mechanical engineering. There is an urgent need to systematically integrate and sort out the existing achievements, so as to clarify the development context and future directions of the field [6].

In the realm of energy conversion and heat transfer enhancement, the multi-field coupling properties of ferrofluids have opened up a novel pathway for improving energy utilization efficiency. Bezaatpour et al. integrated a rotary absorber tube, a magnetic field inducer, and nanofluid, achieving a 37.4% reduction in total heat loss from a parabolic trough solar collector while increasing the performance evaluation criterion by 101%. This study confirmed the heat transfer enhancement effect of multi-physical field synergy [14]. Subsequent research has further advanced this direction: Munir et al. investigated the chemically reactive flow of micropolar Eyring–Powell ferrofluid over a stretching surface, uncovering the regulatory mechanisms by which magnetic field and chemical reaction coupling influence velocity and temperature fields [15]; Tumse et al. demonstrated through numerical simulations that a non-uniform magnetic field can maximize the average Nusselt number of ferrofluid around a confined cylinder by 23.26%, providing a theoretical basis for the design of magnetically controlled heat dissipation systems [16]; Barzegar et al. verified in a screw tube that magnetic fields can enhance heat transfer efficiency by 26% through the induction of secondary flows [17]; meanwhile, a review by Dawood et al. indicated that the integration of ferrofluids with magnetic fields can maximize solar collector efficiency by up to 47% [18]. The foundational application framework established by these studies paved the way for advanced thermal systems that incorporate thermoreversible sol–gel transitions to regulate heat transfer dynamically.

In the fields of environmental governance and analytical detection, the high dispersibility and controllable separation properties of ferrofluids have been fully leveraged. Anushree et al. prepared Ni-doped iron oxide colloidal nanocrystal clusters that achieved a 99% adsorption rate for methylene blue, retaining a 70% adsorption efficiency even after five regeneration cycles, thus demonstrating excellent reusability [19]. Zheng et al. developed a self-dispersing ferrofluid based on deep eutectic solvents, which enables rapid extraction of chiral fungicides without complex pretreatment. In food detection applications, it achieved a recovery rate ranging from 83.0% to 101.7% [20]. Immobilizing these highly active magnetic nanoparticles within porous hydrogel networks is emerging as a critical strategy to prevent nanoparticle leaching while maintaining the high enrichment factors required for environmental monitoring [13].

In mechanical engineering and interdisciplinary applications, the magnetically tunable characteristics of ferrofluids have driven innovations in device functionality. Liu et al. designed a symmetric-structured ferrofluid sealing device, maximizing sealing performance by optimizing pole tooth parameters, which provides a design basis for small-gap sealing applications [21]. The expansion of this research direction is evident across multiple fields: Jabłońska et al. utilized a ferrofluid-coated, rotating magnetic field-assisted bioreactor to regulate microbial metabolic rates, increasing bacterial growth rates by approximately 30% [22]; Crepaldi et al. discovered that Fe_3_O_4_ water-based ferrofluids exhibit memristive properties, enabling low power consumption (<200 μW) in memory computing functions [23]; Fan et al. designed ferrofluid droplets as liquid microrobots, which can undergo deformations such as elongation and splitting under magnetic field regulation to complete cargo transportation in complex terrains [24]. Embedding these adaptable droplets within soft gel matrices allows for the creation of robust soft robotic actuators. Meanwhile, Li et al. developed a variable-stiffness ferrofluid shock absorber based on an optimized stiffness formula, achieving a vibration attenuation rate of 98.7% and providing a novel solution for spacecraft vibration control [25].

**Figure 1:**
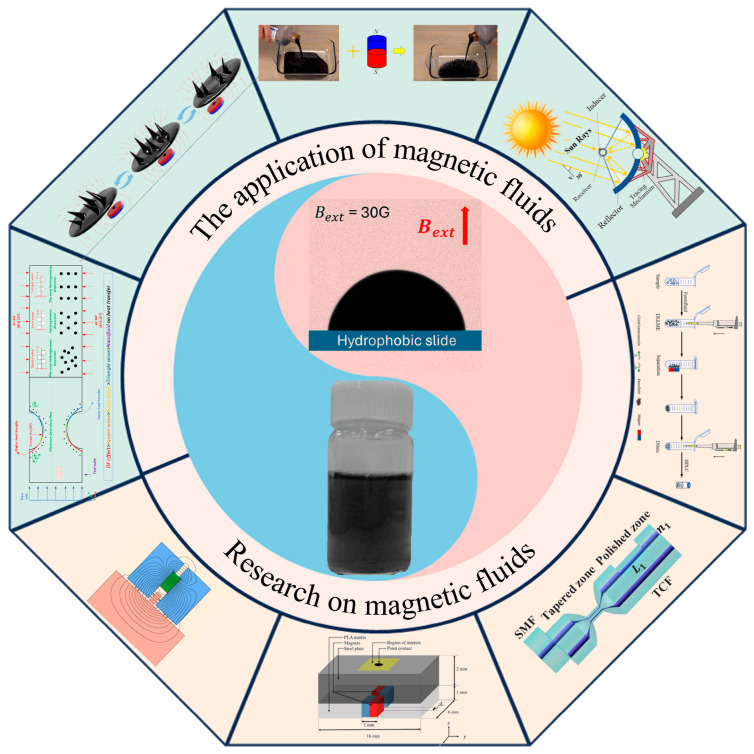
This study provides a comprehensive review of the research on the magnetic fluid robot itself and its applications.

Overall, existing studies have established a relatively complete research system concerning the preparation process optimization, multi-field performance regulation, and multi-scenario application expansion of ferrofluids, yet numerous critical bottlenecks remain to be overcome. Among these, the insufficient long-term stability caused by nanoparticle agglomeration in biomedical applications stands out as the most pressing issue. Cross-linking ferrofluids with three-dimensional polymer networks to construct ferrogel systems can realize the stable immobilization of magnetic particles through the matrix confinement effect, effectively addressing the aforementioned stability drawbacks of pure fluid systems [26]. Although systematic progress has been achieved in the controllable synthesis of magnetic nanomaterials, the clarification of multi-field coupling mechanisms, and the development of multi-domain functional applications, the field still faces a set of common challenges: the long-term stability of magnetic nanomaterials in complex biological media and high-salt engineering environments needs to be enhanced, the dynamic correlation between microstructural evolution and macroscopic properties in fluid–gel composite systems has not been fully elucidated, and there are still distinct barriers to the large-scale preparation and clinical/engineering translation of multi-field responsive multifunctional integrated materials [7,9]. In view of this, this paper comprehensively reviews the established and emerging applications of ferrofluids and their composite magnetic gel systems, with a focus on the latest breakthroughs in biomedicine and engineering over the past five years, and deeply analyzes the inherent logical connections and common technical bottlenecks across different research directions, aiming to provide systematic theoretical references and clear technical guidance for cross-scenario integrated applications and technological innovation in this field (Figure 1).

## 2. Electron Microscopy Investigation

**Figure 2:**
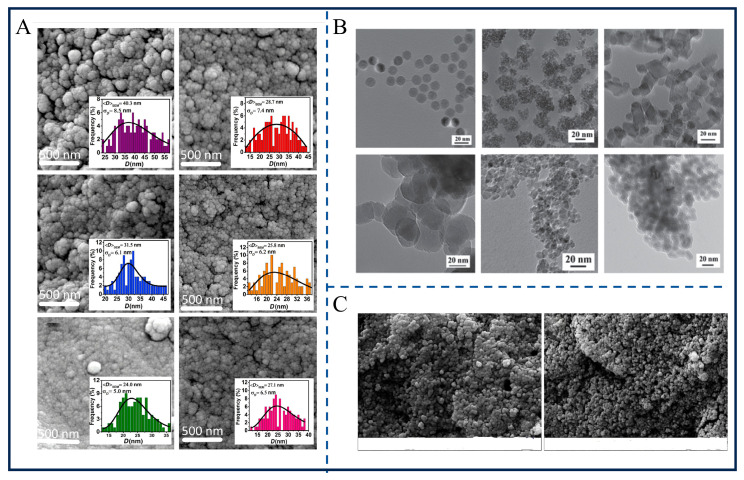
Electron microscope images of different magnetic fluids. (**A**) FE-SEM image of Co_x_Fe_3−x_O_4_ nanoparticles. Reproduced from Ref. [35] with permission from *Beilstein Journal of Nanotechnology*. (**B**) TEM image of MNPs. Reproduced from Ref. [36] with permission from *Advanced Functional Materials*. (**C**) SEM images of CuFe_2_O_4_ NPs and Fe_3_O_4_ NPs. Reproduced from Ref. [41] with permission from *Journal of Alloys and Compounds*.

Magnetic nanomaterials serve as the core functional building blocks of ferrofluid and ferrogel systems. Endowed with unique magnetic properties (e.g., superparamagnetism and high-saturation magnetization) and precisely tailorable surface physicochemical properties, they exhibit substantial application potential in environmental pollutant removal, tumor therapy, biodetection, and other fields [27]. Superparamagnetism is a unique magnetic behavior exhibited by magnetic nanoparticles with a particle size below the critical single-domain size at room temperature: when the thermal motion energy of the particles exceeds their intrinsic magnetocrystalline anisotropy energy, the magnetic moments of the particles can flip freely and randomly. Macroscopically, the material shows no remanence or coercivity in the absence of an external magnetic field, while generating a strong magnetic response upon the application of an external magnetic field [28,29]. This property serves as the core physical basis for ferrofluids to achieve long-term colloidal stability and rapid, controllable response under magnetic fields, as well as the key prerequisite for their applications in scenarios including magnetic hyperthermia and magnetic resonance imaging (MRI). The functional efficiency of magnetic materials is fundamentally governed by the intrinsic correlations between magnetocrystalline anisotropy, interparticle interactions, and macroscopic performance [30,31,32]. To date, a series of key advances have been achieved in the precise structural regulation of materials and the elucidation of structure–property relationships. Through systematic optimization of synthesis processes and surface modification techniques, designable and quantitative control over the core magnetic properties and surface physicochemical characteristics of nanoparticles has been realized [33,34]. This overcomes the technical bottlenecks of conventional magnetic materials, such as single functionality and poor controllability, and lays a solid foundation for their large-scale applications across diverse fields. Crucially, the structural characteristics of these nanoparticles dictate their compatibility and distribution when cross-linked into ferrogel or magnetic hydrogel networks. For instance, in magnetic hyperthermia, materials convert electromagnetic energy into thermal energy through hysteresis loss or relaxation effects under an alternating magnetic field (Jalili et al., Figure 2A) [35]; in immunoassays, surface-functionalized magnetic nanoparticles achieve targeted capture via antibody–antigen specific binding (Zhu et al., Figure 2B) [36]; in adsorption applications, the high specific surface area and magnetic responsiveness of nanostructures enable the efficient separation of pollutants [19]. These applications all depend on the precise regulation of material morphology, size, and magnetic properties, while electron microscopy techniques (Scanning Electron Microscope (SEM), Transmission Electron Microscope (TEM)) serve as key tools to elucidate the structure–property relationship of materials both in continuous fluid phases and within porous gel matrices [37,38].

SEM and TEM have been employed to characterize the morphology and size of magnetic nanomaterials, revealing common features such as nanoscale dimensions and spherical particle characteristics [39,40]. Zhu et al. observed via TEM that magnetic nanoparticles (MNPs) synthesized through the thermal decomposition method are spherical, with sizes ranging from 4 to 20 nm, and particles synthesized using alternative methods (coprecipitation, microemulsion) all exhibit a narrow size distribution [36]. FE-SEM results from Jalili et al. revealed that Co_x_Fe_3-x_O_4_ particles are quasi-spherical, with an average size of 24.0–40.3 nm, with aggregation occurring due to magnetic interactions. TEM and SEM images from Fotukian et al. confirmed that CuFe_2_O_4_ (19.9 nm) and Fe_3_O_4_ (18.5 nm) synthesized using the solvothermal method are monodisperse spherical particles with uniform size distribution (Figure 2C) [41]. Additionally, SEM results of Ni-doped iron oxide colloidal nanocrystal clusters showed that they have an average size of 132–198 nm and exist in a quasi-spherical aggregated state. The differences primarily lie in the correlation between dispersibility and synthesis methods: Co_x_Fe_3-x_O_4_ exhibits obvious aggregation due to dipole–dipole interactions (particle agglomeration observed by SEM), whereas CuFe_2_O_4_ shows significantly improved monodispersity owing to triethylene glycol coating and regulation by the solvothermal method. High monodispersity is particularly advantageous for ferrogel synthesis, ensuring uniform cross-linking and consistent magnetomechanical responses across the gel network. These studies compared electron microscopy results across various synthesis methods and found that the thermal decomposition method is more likely to yield particles with uniform size.

Electron microscopy characterization reveals the direct impact of material structure on functionality. Jalili et al. found via FE-SEM that Co doping increased the size of Fe_3_O_4_ particles from 7.5 nm to 13.1 nm (based on XRD crystallite size), but the agglomeration phenomenon observed in SEM (40.3 nm) correlates with magnetic properties—aggregation enhances interparticle interactions, increasing the difficulty of regulating magnetocrystalline anisotropy and ultimately reducing hyperthermia efficiency (with the specific absorption rate (SAR) decreasing from 1.33 W/g to 0.37 W/g). TEM results from Fotukian et al. showed that the 19.9 nm size and monodispersity of CuFe_2_O_4_ facilitate magnetization reversal in an alternating magnetic field; combined with its low magnetocrystalline anisotropy (6.25 × 10^5^ erg/cm^3^), its SAR value reaches 44.9 W/g, more than twice that of Fe_3_O_4_ (18.5 W/g).

The Vibrating Sample Magnetometer (VSM) is a core tool for the quantitative characterization of the magnetic response performance of magnetic nanoparticle and ferrogel systems. The saturation magnetization (*M_s_*) obtained from VSM measurements is a key parameter that determines the magnetothermal efficiency, magnetically actuated performance, and magnetorheological properties of the materials [42]. VSM characterization results from existing studies show that monodisperse pure-phase Fe_3_O_4_, CoFe_2_O_4_, and CuFe_2_O_4_ nanoparticles with superparamagnetic sizes typically exhibit a saturation magnetization of 30–80 emu/g at room temperature, with no obvious remanence or coercivity, thus maintaining typical superparamagnetic characteristics [43,44,45]. When the aforementioned nanoparticles are encapsulated into the three-dimensional polymer network of hydrogels via physical doping or chemical crosslinking, the saturation magnetization of the obtained ferrogels decreases by 10–40% compared with that of the pure-phase nanoparticles [44,46]. This variation is mainly regulated by two core mechanisms: The first is the non-magnetic dilution effect of the polymer matrix. The mass fraction of magnetic particles in the gel system is lower than that in the pure powder material, which reduces the total magnetic moment per unit volume and directly leads to a decrease in macroscopic saturation magnetization. The second mechanism is the steric hindrance effect of the polymer chains. The physical confinement effect of the gel network hinders the free rotation of the magnetic moments of the nanoparticles under an external magnetic field, which further weakens the macroscopic magnetic response capability of the material [47].

It is worth noting that polymer encapsulation does not alter the intrinsic superparamagnetic properties of the nanoparticles, and the ferrogels still retain the core advantages of zero remanence and rapid magnetic response [43]. Meanwhile, the three-dimensional hydrogel network can effectively inhibit the agglomeration of nanoparticles in complex body fluids and high-salt environments, ensuring the long-term stability of the saturation magnetization and magnetothermal performance of the materials during repeated cycles. This is also the core advantage of ferrogels over pure ferrofluids in biomedical and long-cycle engineering applications. A comparison of VSM parameters between typical magnetic nanoparticles and their corresponding ferrogels, along with the relevant references, is summarized in Table 1.

This chapter systematically reviews the electron microscopy characterization methods, structural characteristics, and structure–performance relationships of magnetic nanomaterials. Based on existing research findings, the field of structural characterization for magnetic nanomaterials has attained multi-dimensional landmark progress. The electron microscopy characterization system using SEM and TEM as its core has been well developed, which enables the multi-scale and precise characterization of the morphology, size, dispersibility and microcrystalline structure of magnetic nanomaterials and forms a standardized analytical methodology [48,49]. Meanwhile, breakthroughs have been achieved in the combined technology of in situ electron microscopy and magnetic property measurement, which can accurately capture real-time structural changes in materials and their surrounding gel matrices in practical application environments [50]. Furthermore, the aggregation mechanism of nanoparticles in complex biological environments needs to be further explored using electron microscopy–spectroscopy coupling techniques to clarify the function of surface coating layers, so as to provide more accurate structural guidance for the design of high-efficiency and stable magnetic nanomaterials for hydrogel encapsulation.

## 3. Peak State Characteristics

Ferrofluids, as colloidal suspensions of magnetic nanoparticles, have macroscopic behaviors (e.g., deformation, migration, and phase transitions) and microscopic mechanisms (e.g., dipolar chain formation and interfacial tension changes) that are at the core of research in the field of ferrohydrodynamics. Under the influence of an external magnetic field, these colloidal suspensions often exhibit profound magnetorheological gelation, temporarily transitioning from a fluid “sol” state to a highly structured, viscoelastic gel-like state. Rheological characterization is the most direct method for quantifying the magnetorheological gelation process of ferrofluids under external magnetic fields [51]. The sol–gel transition of ferrofluids is essentially a transformation from a viscous fluid state to an elastic gel state, which can be clearly characterized by the variation patterns of the storage modulus (G′, which characterizes the elastic properties of materials) and loss modulus (G″, which characterizes the viscous properties of materials). For a monodisperse ferrofluid system, in the absence of an external magnetic field, G″ is significantly higher than G′, and the system exhibits typical Newtonian fluid characteristics, namely the sol state. As the external magnetic field strength increases, the magnetic dipole–dipole interactions between nanoparticles are enhanced, and the nanoparticles gradually assemble to form chain-like and network-like microstructures, leading to a rapid increase in G′. When the magnetic field strength reaches the critical threshold, G′ exceeds G″, which marks the completion of the sol–gel transition and the transformation of the system into a viscoelastic gel state. A further increase in magnetic field strength will continuously enhance the crosslinking density of the dipole network, resulting in a sustained rise in G′ and the elastic modulus of the gel [52,53,54]. Studies have revealed that non-uniform magnetic fields can induce shape and fission instabilities in ferrofluids; the deformation of hydrophobic droplets in uniform magnetic fields is regulated by apparent interfacial tension [55]; the formation of dipolar chains dominates microstructure evolution, providing the physical cross-linking framework necessary for this transient gelation [56]; and numerical simulation methods (such as the conservative level set method) enable the accurate capture of droplet coalescence dynamics [57]. Additionally, rotating magnetic fields can drive droplet wobbling and migration [58]; weavable ferrofluid fibers can realize electromagnetic early warning functions [59]; and droplet wettability can be reversed under supersaturated magnetic fields [60]. These investigations have laid a foundational understanding for the interaction between magnetic fields and ferrofluids.

**Figure 3:**
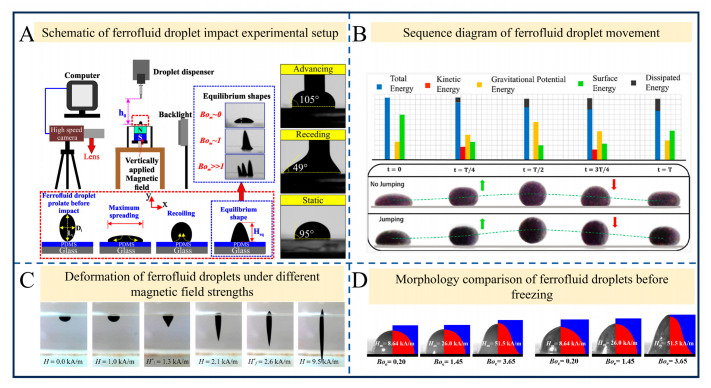
Magnetic fluid peak characteristic diagram. (**A**) Schematic diagram of the experimental setup used to study the phenomenon of iron magnetic fluid droplet impact. Reproduced from Ref. [61] with permission from *Colloids and Surfaces A: Physicochemical and Engineering Aspects*. (**B**) Sequence diagram of magnetic fluid droplet movement. Reproduced from Ref. [62] with permission from *LANGMUIR*. (**C**) Deformation of FF 3 drop with the growth of magnetic field intensity H. Reproduced from Ref. [63] with permission from *MethodsX*. (**D**) Comparisons of the shapes of ferrofluid droplets before freezing. Reproduced from Ref. [64] with permission from *Physical Review Fluids*.

A core issue in this field is the dynamic response mechanism of these fluid–gel systems. As illustrated in Figure 3A, Shyam et al. utilized high-speed imaging to observe that, when a ferrofluid droplet impacts a PDMS (polydimethylsiloxane) substrate under a non-uniform magnetic field [61], its equilibrium morphologies (no spike, single spike, and multiple spikes) are jointly governed by the Weber number (ratio of inertial force to surface tension) and the magnetic Bond number (ratio of magnetic force to surface tension). The formation of nanoparticle chains increases viscosity, resulting in an inverse relationship between the spreading diameter and magnetic field strength. As shown in Figure 3B, Mohammadrashidi et al. explored the vibration and jumping behaviors of ferrofluid marbles: the vibration of marbles under magnetic perturbation can be modeled as a mass–spring–damper system, where the natural frequency decreases with increasing volume and the damping ratio exhibits a linear relationship with the Ohnesorge number (ζ = 6.22 Oh) [62]. This viscoelastic behavior is highly characteristic of soft gel materials. When magnetic deformation is sufficiently large, marbles jump due to the conversion of surface energy into gravitational potential energy.

Notable breakthroughs have also been made in measurement methods and phase transition behaviors. Khokhryakova et al. proposed a new interfacial tension measurement method: a ferrofluid droplet elongates in a vertical magnetic field, and the ratio of the critical magnetic field strengths governing the instability of the liquid–liquid interface and that of the free surface equals the square root of the ratio of their surface tensions (Figure 3C) [63]. This non-contact method has a deviation of less than 10% from the results of the traditional ring method, solving the problem of difficult measurement of interfacial tension in magnetic fluids. As shown in Figure 3D, Fang et al. found through numerical simulations that magnetic field squeeze causes droplet flattening (the height decreases linearly under low magnetic fields and follows a $Bo_{e}^{-0.5}$ relationship under high magnetic fields), while magnetic field lift leads to elongation [64]. Moreover, the propagation of the freezing front obeys the $h∼t^{0.5}$ law under high squeeze magnetic fields—a process closely mirroring the thermodynamic solidifications observed in thermosensitive hydrogels.

Existing studies have elucidated the laws governing the magnetic field regulation of ferrofluids from multiple dimensions, yet several directions remain to be explored. For instance, the real-time correlation between microstructures (e.g., dipolar chains) and macroscopic behaviors (e.g., impact spikes) during dynamic processes remains poorly understood, calling for the integration of in situ characterization techniques (e.g., the combination of cryogenic electron microscopy and high-speed imaging). In summary, research on the magnetic field regulation of ferrofluids must continue to achieve breakthroughs in micro–macro correlation, multi-field coupling modeling, and practical application technologies to advance its applications in fields such as microfluidic manipulation and stimuli-responsive smart ferrogels.

## 4. Preparation of Magnetic Fluid and Ferrogel Systems

Ferrofluid, a colloidal suspension composed of magnetic nanoparticles dispersed in a carrier liquid, has demonstrated significant potential in engineering technologies and biomedicine owing to its unique combination of magnetic responsiveness and fluidity. The core of ferrofluid preparation—and its subsequent transition into stable ferrogel systems—lies in achieving stable dispersion and functional regulation of particles. First, magnetic nanoparticles (e.g., Fe_3_O_4_, CoFe_2_O_4_) are synthesized using methods like chemical co-precipitation, hydrothermal synthesis, or sol–gel transitions. Then, surfactants or ligand exchange strategies are employed to enhance particle dispersion. Finally, these modified particles are integrated with carrier liquids to form ferrofluids, which can subsequently act as precursors that cross-link with polymer networks to form solid or semi-solid magnetic hydrogels [65].

**Figure 4:**
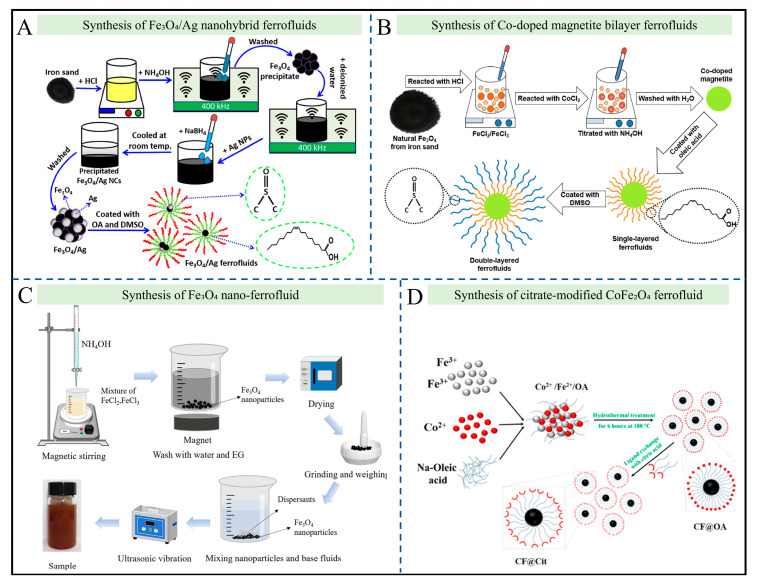
Schematic diagram of the preparation process of different magnetic fluids. (**A**) Synthesis scheme of the Fe_3_O_4_/Ag nanohybrid ferrofluids. Reproduced from Ref. [66] with permission from *Heliyon*. (**B**) Illustration of the formation of the co-doped magnetite double-layered ferrofluids. Reproduced from Ref. [67] with permission from *Journal of King Saud University—Science*. (**C**) Fe_3_O_4_ nano-ferrofluid preparation method. Reproduced from Ref. [68] with permission from *Journal of Cleaner Production*. (**D**) Schematic of the synthesis of oleic acid and citric acid modified cobalt ferrite ferrofluid. Reproduced from Ref. [69] with permission from *Colloids and Surfaces A: Physicochemical and Engineering Aspects*.

Ferrofluid preparation can be categorized into two types based on raw material sources and basic synthesis routes. The green synthesis strategy, which utilizes natural iron sand as the raw material, offers the advantages of having a low cost and high environmental friendliness: Taufiq et al. used Fe_3_O_4_ extracted from iron sand as the core, combined it with Ag nanoparticles via a hydrothermal method to form Fe_3_O_4_/Ag nanohybrid particles, and then obtained stable nanohybrid ferrofluid by applying a double-layer coating (oleic acid as the first layer and DMSO (Dimethyl Sulfoxide) as the second layer) and dispersing the particles in olive oil (Figure 4A) [66]. The crystallite sizes of Fe_3_O_4_ and Ag in this ferrofluid are 11.8–12.1 nm and 36.8–37.2 nm, respectively. Similarly, as shown in Figure 4B, Taufiq et al. also used iron sand as the raw material to prepare Co-doped magnetite particles (size 7–12 nm) through Co^2+^ doping, and dispersed them in olive oil using the same double-layer coating strategy (oleic acid + DMSO) [67]. The lattice parameter expanded from 8.355 Å to 8.422 Å as the Co^2+^ content increased. The other category is chemical reagent synthesis, which focuses on the precise regulation of particle size, composition, and network structure. Xing et al. synthesized Fe_3_O_4_ nanoparticles (with a diameter of 12.61 nm) via chemical co-precipitation, using a water–ethylene glycol mixture (4:1) as the base liquid (Figure 4C) [68]. Notably, sol–gel synthesis has emerged as a highly controllable method within this category. This technique involves the transition of a system from a liquid “sol” (mostly colloidal) into a solid “gel” phase. In conventional fluid systems, researchers have synthesized Fe_3_O_4_ nanoparticles via chemical co-precipitation using a water–ethylene glycol mixture. In sol–gel methodologies, these nanoparticle sols undergo controlled hydrolysis and polycondensation, creating a rigid, three-dimensional silica or polymer matrix that securely encapsulates the magnetic particles. Furthermore, as shown in Figure 4D, Duraisamy et al. prepared CoFe_2_O_4_ ferrofluid through a two-step method: first, forming hydrophobic particles via oleic acid coating; then, obtaining hydrophilic citrate-modified particles (size 9 ± 4.3 nm) through citrate–oleic acid ligand exchange, which were dispersed in water for magnetic hyperthermia research [69].

Surface modification and functionalization strategies form another important classification dimension, with the core goal of improving stability, endowing specific functions, and facilitating gelation. Double-layer coating technology is widely used to enhance ferrofluid stability: the Taufiq team leveraged the synergistic effect of oleic acid and DMSO. The hydrophobic moieties of oleic acid anchor to the particle surface, while the polar groups of DMSO improve compatibility with the carrier liquid, effectively inhibiting particle agglomeration and providing functional sites for subsequent biological applications. The ligand exchange method focuses on functional conversion: Duraisamy et al. converted hydrophobic CoFe_2_O_4_ particles to hydrophilic ones through ligand exchange between citrate and oleic acid, resulting in a zeta potential of −27 mV [69]. This not only ensures colloidal stability but also enhances compatibility with the biological environment, laying the foundation for cellular internalization and tumor therapy. The dispersant-assisted method is optimized for specific application scenarios: Xing et al. added a dispersant to the water–ethylene glycol base liquid to ensure the uniform dispersion of Fe_3_O_4_ particles. Its optical absorption peaks (at 220 nm, 500–600 nm, and 970 nm) match the solar spectrum, making it suitable for photothermal conversion requirements.

Zn_x_Mn_1−x_Fe_2_O_4_ mixed spinel ferrites are key magnetic materials widely applied in magnetic hyperthermia and magnetic resonance imaging (MRI). Their magnetocrystalline anisotropy, saturation magnetization (M_s_) and superparamagnetic properties can be precisely regulated by tuning the Zn/Mn stoichiometric ratio *x*. The mainstream preparation routes for this system are dominated by wet chemical methods, supplemented by solid-phase mechanochemical approaches [70]. Chemical co-precipitation, the most common precursor synthesis method for such ferrofluids, boasts simple operation, mild conditions and easy industrial scale-up [71]. Hydrothermal/solvothermal synthesis yields products with high crystallinity, narrow size distribution and excellent monodispersity, enabling continuous grain size tuning from 7 to 60 nm, highly controllable magnetic properties and markedly improved long-term dispersion stability of ferrofluids. The sol–gel method achieves atomic-level homogeneous mixing of multi-metal elements, delivering products with outstanding compositional uniformity and a broad tunable range of magnetic properties for high-precision applications. Solid-phase methods like high-energy ball milling feature short processes, solvent-free production and scalability, yet tend to introduce lattice stress and impurities, with a far inferior product controllability compared to wet chemical methods [72].

This chapter systematically summarizes the controllable preparation methods, surface functionalization modification strategies, and regulation mechanisms of the sol–gel transition for ferrofluid and ferrogel systems. Based on existing research achievements, the field has yielded landmark research advances. The synthetic routes have been diversely expanded, forming two major technical systems: low-cost green synthesis using natural iron ore as a raw material, and chemical synthesis enabling the precise control of particle size, composition, and magnetic properties, which can fully meet the material requirements of diverse application scenarios. The surface modification technology system has become increasingly mature, with a variety of universal modification strategies developed, including double-layer coating, ligand exchange, and dispersant-assisted modification, which effectively solve the common industrial problems of magnetic nanoparticles, such as easy agglomeration and poor dispersion stability [73,74,75,76]. The sol–gel synthesis technique has achieved precise controllability: via the controlled hydrolysis and polycondensation of nanoparticle sols, rigid three-dimensional silica or polymer matrices can be constructed to stably encapsulate magnetic particles, providing core technical support for the controllable preparation of ferrogels. A key breakthrough has been made in the in situ crosslinking technology for ferrogels, which can efficiently crosslink surface-modified magnetic nanoparticles with polymer hydrogel networks, realizing the trans-morphological functionalization from liquid ferrofluids to solid magnetic hydrogels and fundamentally resolving the prevalent issue of particle loss in pure fluid systems for biomedical and engineering applications [77,78]. Currently, the field still suffers from drawbacks including an insufficient standardization of large-scale preparation processes for multifunctional composite ferrogels, poor controllability during the crosslinking of multi-component systems, and an imperfect preparation system for biomedical-grade materials, which represent the core directions requiring key breakthroughs in the subsequent research and development of preparation technologies.

## 5. Application in the Medical Field

In this section, we will present a detailed overview of the applications of ferrofluids and their integration into magnetic gel matrices in the medical field, as well as the latest research progress. The discussion will center on three key aspects: (i) hyperthermia therapy, (ii) targeted drug delivery to specific sites, and (iii) medical imaging. Integrating ferrofluids into ferrogels (magnetic hydrogels) offers distinct advantages in these areas by preventing nanoparticle diffusion in vivo and enabling controlled, stimuli-responsive behaviors.

### 5.1. High-Temperature Hyperthermia

Tumor hyperthermia, as a minimally invasive therapeutic approach, induces cancer cell apoptosis by elevating the temperature of the tumor region to 42–46 °C while minimizing damage to normal tissues. Its core lies in utilizing functional materials to achieve precise temperature control and targeted delivery. Magnetic nanoparticles (such as iron oxide and manganese ferrite) generate heat through Néel relaxation or Brown relaxation under an alternating magnetic field (Bellizzi et al.) [79]. Thermosensitive liposomes and thermo-responsive magnetic hydrogels release drugs at specific temperatures [80], while near-infrared light-responsive materials produce heat via photothermal conversion [81]. The design and optimization of these materials represent key research directions.

**Figure 5:**
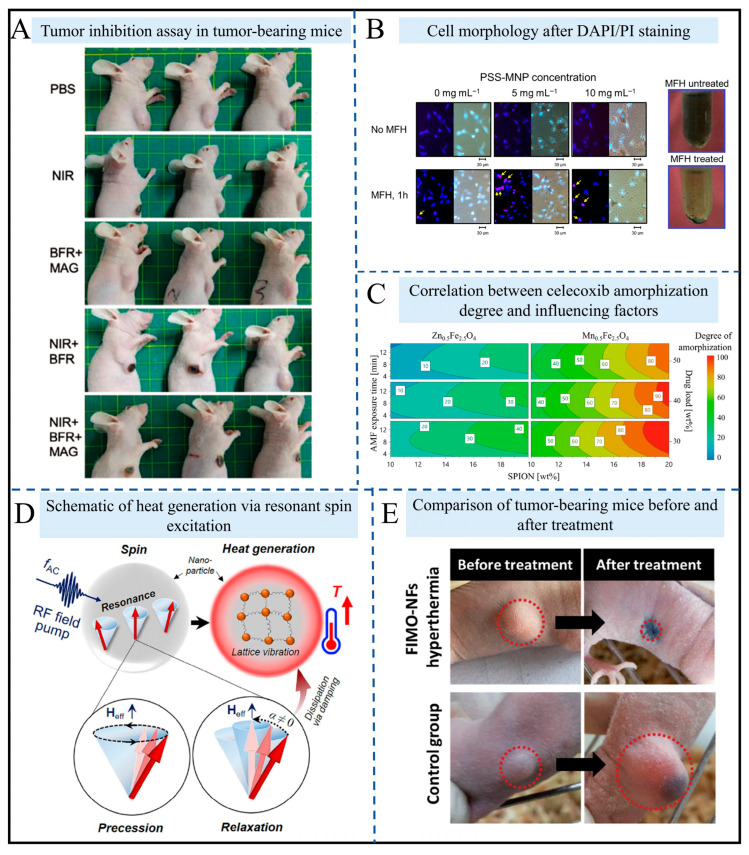
High-temperature hyperthermia function diagram. (**A**) Tumor-killing experiment in mice, showing the tumor status of each experimental group of nude mice six days after treatment. Reproduced from Ref. [81] with permission from *Advanced Healthcare Materials*. (**B**) Cell morphology and appearance of cells stained with DAPI/PI. Reproduced from Ref. [82] with permission from *PLoS ONE*. (**C**) Contour plot shows the relationship between the degree of solubilization of celecoxib amorphous form containing Zn_0.5_Fe_2.5_O_4_ and Mn_0.5_Fe_2.5_O_4_ and the exposure time of AMF, the content of doped SPION and the drug loading. Reproduced from Ref. [83] with permission from *ACS Applied Materials & Interfaces*. (**D**) Resonant spin excitation and relaxation dynamics for dissipating local heating. The energy generated by magnetic loss is dissipated in the form of lattice vibration through various spin–lattice interactions, thereby increasing the temperature of magnetic nanoparticles. Reproduced from Ref. [84] with permission from *Scientific Reports*. (**E**) Representative mice from the two groups on the 50th day: FIMO-NFs hyperthermia treatment group and control group. Reproduced from Ref. [86] with permission from *Advanced Healthcare Materials*.

As shown in Figure 5A, the biocompatible ferrofluid-based millirobot (BFR) developed by Ji et al. has demonstrated potential for targeted therapy: using corn oil as the matrix and oleic acid-coated Fe_3_O_4_ nanoparticles, it achieves precise 3D movement under the control of a four-coil magnetic field system (with an error of 0.1 mm) [81]. These liquid robotic systems are highly complementary to soft ferrogel robots, where nanoparticles are trapped in a cross-linked elastomer or gel, offering structural stability while maintaining photothermal capabilities. Under 1064 nm near-infrared light irradiation, it heats up to 49 °C within 5 min, and the inhibition rate of 4T1 tumors in tumor-bearing mice reaches 67.48%. Polystyrene sulfonic acid-coated magnetic nanoparticles (PSS-MNPs) were developed by Chen et al., with a focus on optimizing hyperthermia parameters [82]. It was found that extracellular hyperthermia (5 mg/mL, 43.7 kHz) applied for 3 h can reduce the survival rate of SK-Hep1 cells to 33.71%, while reducing the culture medium volume (to 0.2 mL) or adopting intermittent treatment (three cycles) can mitigate heat diffusion.

As shown in Figure 5C, Ansari et al. proposed a novel application for superparamagnetic iron oxide nanoparticles (SPIONs): Mn_0.5_Fe_2.5_O_4_ nanoparticles generate heat under an alternating magnetic field, enabling in situ amorphization of celecoxib in oral tablets, which increases its solubility by 5-fold [83], thus addressing the drawback of poor stability in traditional amorphous preparations. This solid-state entrapment mirrors the encapsulation process used in drug-loaded ferrogels, preventing drug recrystallization. The resonant spin mechanism discovered by Lee et al. breaks through the efficiency bottleneck of conventional hyperthermia (Figure 5D) [84]. Fe_3_O_4_ nanoparticles achieve an initial temperature rise rate of 150 K/s under a 3.0 GHz microwave magnetic field, providing a new principle for ultra-rapid hyperthermia—similar to how cobalt-doped nanoparticles enhance heat production through magnetocrystalline anisotropy [85]. The Fe_0.6_Mn_0.4_O nanoflowers developed by Liu et al. integrate T1-T2 dual-mode MRI (r1 = 4.9 mM^−1^s^−1^, r2 = 61.2 mM^−1^s^−1^) with magnetic hyperthermia, boasting a specific absorption rate (SAR) of 535 W/g. They enable the clear visualization and complete elimination of tumors in glioma models (Figure 5E) [86].

The current research exhibits three major trends: first, multifunctional integration, such as nanoflowers combining diagnosis and treatment, and BFR integrating magnetic control with photothermal therapy; second, mechanism innovation, where resonant spin and in situ amorphization overcome traditional technical bottlenecks; third, orientation toward clinical translation, emphasizing the balance between safety and effectiveness from material biocompatibility to parameter optimization. In the future, it will be necessary to address issues such as long-term material toxicity and the adaptation of individualized treatment parameters to advance hyperthermia from basic research to routine clinical application.

### 5.2. Targeted Drug Delivery

Magnetic materials have demonstrated significant potential in fields such as biomedicine and flexible robotics due to their controllable responsiveness to magnetic fields. The core principle revolves around the interaction between magnetic fields and materials: regulating the morphology, movement, or release behavior of materials through magnetic field forces, while integrating the intrinsic physical and chemical properties of the materials (e.g., interfacial tension, coordinate bonds, magnetothermal effects) to achieve functions such as targeted delivery, precise therapy, or environmental adaptation [87]. For example, the encapsulation of ferrofluids leverages the competition between magnetic field forces and interfacial tension to form stable core–shell structures [88]. When cross-linked, these structures form robust magnetic microgels capable of localized therapy.

**Figure 6:**
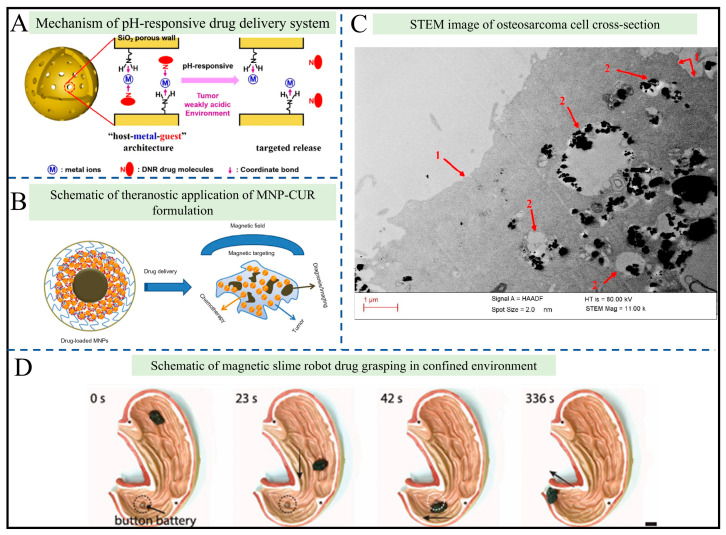
Schematic diagram of magnetic fluid targeted drug delivery function. (**A**) Schematic mechanism for the pH-responsive drug delivery system based on the coordination bonding in the mesochannels of HMSNs-NH2. Reproduced from Ref. [89] with permission from *ACS Omega*. (**B**) Schematic representation for theranostic application of MNP-CUR formulation. Reproduced from Ref. [90] with permission from *International Journal of Nanomedicine*. (**C**) STEM image of the cross-section of an osteosarcoma cell with Fe_67.2_Cr_12.5_Nb_0.3_B_20_ magnetic particles after 24 h of co-incubation. Reproduced from Ref. [91] with permission from *Nanomaterials*. (**D**) Slime grasps target objects in complex restricted environment in endocytosis mode. Reproduced from Ref. [92] with permission from *Advanced Functional Materials*.

In the field of drug delivery, environmental responsiveness and targeting mechanisms are key research focuses. Magnetic hydrogels are highly valued here, as their sol–gel phase transitions can be engineered to respond to specific biological triggers. Deng et al. constructed a pH-responsive hollow mesoporous silica nanocarrier system (Figure 6A) [89]. Through a “host–metal–guest” coordination structure (involving coordinate bonds between amino-functionalized carriers, Cu^2+^, and doxorubicin), drugs are released in the weakly acidic tumor microenvironment (pH 5.0–6.4) due to proton competition for coordinate bonds. The release rate reaches nearly 100% at pH 5.0, with a drug loading capacity of 91.8 mg/g, enabling specific targeted release to tumor cells. Similar pH-responsive gelation and degradation mechanics are widely employed in hydrogel networks. As shown in Figure 6B, Yallapu et al. developed curcumin-loaded magnetic nanoparticles (MNP-CUR) that integrate magnetic targeting and theranostic functions: with iron oxide as the core and β-cyclodextrin and Pluronic F68 as coatings, they not only enhance accumulation at tumor sites via magnetic fields but also achieve sustained release of curcumin (47% release over 28 days) [90]. Additionally, they possess MRI contrast capability (T2 relaxivity of 7.04 s^−1^μg^−1^mL), realizing the integration of “diagnosis and treatment”. Although the Fe-Cr-Nb-B ferrofluid developed by Minuti et al. does not directly load drugs, it destroys cancer cells through magnetothermal effects (heating to 45 °C under an alternating magnetic field) and magnetomechanical effects (rotating magnetic fields inducing particle rotation), providing a synergistic killing method complementary to drug delivery and reducing tumor cell survival rates to 25% (Figure 6C) [91].

Mechanical delivery and multifunctional expansion have further enriched the forms of drug delivery. As shown in Figure 6D, the magnetic slime robot developed by Sun et al. [92], relying on non-Newtonian fluid properties, can grasp solid drugs through a curling mode or wrap harmful substances via an endocytosis-like mode. This slime effectively acts as a dynamic gel system. It moves flexibly in narrow channels (1.5 mm in diameter) and across complex interfaces (water, air, and various substrates) to achieve physical drug transport. Its self-healing and conductive properties also enable auxiliary monitoring and repair functions, showcasing the immense potential of soft, gel-like magnetic actuators in vivo.

Existing studies have addressed the limitations of traditional drug delivery, such as poor targeting and weak environmental adaptability, yet challenges persist: the long-term biocompatibility of nanoparticles (e.g., NdFeB particles require SiO_2_ coating modification), control of material stability in large-scale production, and difficulties in integrating multi-technical collaboration (e.g., combined response to pH and magnetic fields). Future efforts can focus on designing multi-stimuli-responsive systems and optimizing the synergy mechanism between robots and carriers to further improve delivery efficiency and safety, facilitating the translation of magnetic materials from laboratory research to clinical applications.

### 5.3. Medical Imaging

In the field of tumor diagnosis and treatment, nanomaterial-based “theranostic” strategies have emerged as a research hotspot, as they enable the synergy of diagnosis, therapy, and efficacy monitoring [93]. The core principle lies in leveraging the unique physicochemical properties of nanocarriers. For example, the magnetothermal effect of magnetic nanomaterials can achieve localized hyperthermia; thermosensitive carriers (including thermo-responsive ferrogels) can enhance chemotherapeutic targeting through temperature-controlled drug release at specific sites; meanwhile, the magnetic relaxation properties or radioactive labeling of nanomaterials can assist in medical imaging [85].

**Figure 7:**
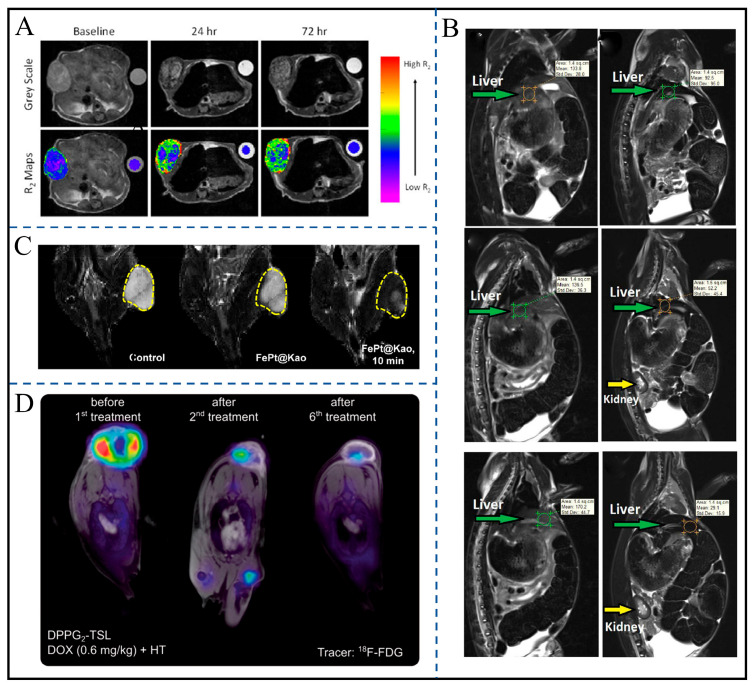
Medical imaging images from different magnetic fluid experiments. (**A**) Representative T 2 -weighted MRI slices, with addition of R 2 maps to aid in the visualization of the observed change in contrast, for PNF treated group in the 18269 model at baseline and at 24 and 72 h post-injection (left to right, respectively) at a fixed TE value. Reproduced from Ref. [94] with permission from *Acta Biomaterialia*. (**B**) Weighted image of the T_2_-injected animal. Reproduced from Ref. [95] with permission from *Materials Chemistry and Physics*. (**C**) In vivo MRI T2-weighted imaging of mice after injection with 100 μL of FePt@Kao in PBS (concentration of 10 mg/mL). Transverse images of a mouse tumor before injection and after injection. Reproduced from Ref. [96] with permission from *Chemistry of Materials*. (**D**) PET/MRI fusion images demonstrating tumor response after two and after six treatments with DOX 0.6 mg/kg in DPPG_2_-TSL-DOX. Reproduced from Ref. [97] with permission from *International Journal of Hyperthermia*.

The preparation and stability of ferrofluids form the foundation for their clinical application, and surface modification technology is key to enhancing their performance. As shown in Figure 7A, McKiernan et al. conjugated PEG chains to the surface of multicore iron oxide nanoflowers (NFs) via a catechol-derived grafting method. The formed hydrated layer allowed the nanoflowers to stably disperse in buffers and biological media for several weeks, with the PEG chain length (2–20 kDa) having no impact on their magnetic properties [94]. Similarly, Vuong et al. modified Fe_3_O_4_ nanoparticles using PMAO as a phase transfer ligand. The resulting ferrofluid exhibited a zeta potential consistently below −40 mV and maintained colloidal stability for up to 6 months, far exceeding that of similar materials modified with SDS or PAA (Figure 7B) [95]. In another approach, Chan et al. modified kaolinite with CTAB, dispersing its layered structure to the nanoscale, which provided a stable loading space for FePt nanoparticles. Through electrostatic and steric hindrance effects, aggregation was reduced, and the targeted accumulation efficiency of the composite material in vivo was improved (Figure 7C) [96]. These studies demonstrate that rational surface modification can address the aggregation issue of ferrofluids by enhancing steric repulsion or hydration, laying the groundwork for the exertion of their imaging and hyperthermia performance.

In medical imaging, the relaxation properties and imaging enhancement effects of ferrofluids are core indicators, with different materials exhibiting unique advantages. McKiernan et al.’s PEGylated nanoflowers, due to the synergistic effect of internal magnetic moments, achieve an r_2_ value of 320 mM^−1^s^−1^. Furthermore, they can stably retain their relaxation performance in a pancreatic cancer model, enabling T_2_-weighted imaging of tumors [94]. Chan et al.’s FePt@Kao composite material, through the synergy between the strong magnetic moments of FePt and the high dispersibility of kaolinite, achieves an r_2_ value of 29.32 mM^−1^s^−1^. It can clearly distinguish tumors from normal tissues in a hepatocellular carcinoma model and supports magnetically guided precise localization [96]. Vuong et al.’s PMAO-modified Fe_3_O_4_ ferrofluid, in in vivo rabbit experiments, increased the T_2_ signal intensity of liver tissue by 5.85 times, with signal enhancement showing a positive correlation with concentration, enabling the quantitative evaluation of nanoparticle accumulation at tumor sites [95]. Additionally, although Zimmermann et al. did not use ferrofluids, they monitored volume changes in feline soft tissue sarcomas after thermosensitive liposome treatment using MRI and ^18^F-FDG PET, indicating that multimodal imaging can complementarily verify therapeutic efficacy and provide auxiliary insights for ferrofluid-based imaging (Figure 7D) [97].

Current research has achieved breakthroughs in the stability, imaging performance, and therapeutic synergy of ferrofluids, yet several directions warrant further exploration. For instance, long-term in vivo metabolic pathways remain to be clarified. In the future, multi-material composite strategies could be explored—such as combining the temperature-responsive properties of thermosensitive liposomes with the imaging–hyperthermia functions of ferrofluids—to achieve closed-loop regulation of “temperature-triggered release–magnetothermal enhancement–imaging monitoring,” thereby promoting the translation of nanotheranostic systems from animal experiments to clinical applications.

## 6. Application in the Field of Engineering

In this section, we will present a detailed overview of the applications of ferrofluids in the engineering field as well as the latest research progress. The discussion will focus on the following aspects: (i) extraction, (ii) friction power generation and solar–thermal/optical conversion, (iii) optical fiber sensors, (iv) heat transfer research, (v) magnetic sealing, and (vi) other applications. Crucially, by cross-linking these functional fluids into soft ferrogel elastomers or utilizing their sol–gel phase transitions, many of these engineering applications gain enhanced mechanical stability, precise structural control, and leak-proof characteristics.

### 6.1. Extraction

As an efficient separation and enrichment technique, ferrofluid extraction has demonstrated significant advantages in recent years across fields such as environmental monitoring, food safety, and biomedicine [98,99]. Its core lies in the synergistic effect between magnetic nanomaterials and novel solvents. To prevent the loss of nanomaterials in the sample and improve reusability, these magnetic nanoparticles are increasingly immobilized within porous hydrogel networks, creating magnetic gel absorbents. Novel solvents (e.g., DES (deep eutectic solvents)) enhance affinity for target analytes and system dispersion by regulating physicochemical properties such as polarity and hydrogen bonding capacity. This technology is primarily divided into two directions: liquid–liquid extraction and liquid–solid extraction. In liquid–liquid extraction, ferrofluid acts as the extraction phase dispersed in the sample solution, with target analytes partitioning between the two phases through hydrophobic interactions, hydrogen bonding, etc. In liquid–solid extraction, magnetic particles are surface-functionalized (e.g., with carbon quantum dots or tannic acid) to adsorb target analytes, while solvents assist particle dispersion to expand the contact area. Finally, particles are recovered via a magnetic field, and target analytes are eluted. This dual mechanism of “magnetic separation + solvent regulation” provides an innovative solution for the efficient pretreatment of trace analytes in complex matrices [13].

**Figure 8:**
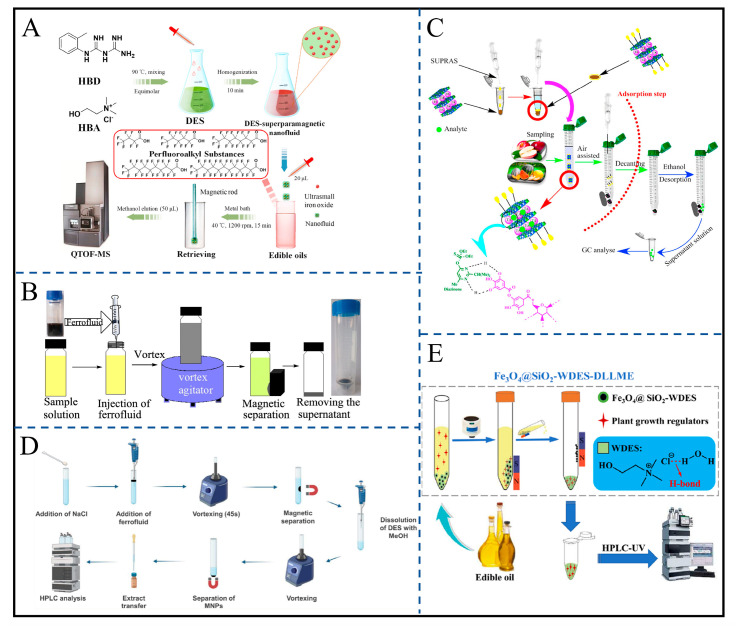
Schematic diagram of different magnetic fluid extraction processes. (**A**) Pretreatment procedure by using deep eutectic solvent-based superparamagnetic nanofluid. Reproduced from Ref. [100] with permission from *Talanta*. (**B**) Carbon quantum dot-modified ferrofluid for dispersive solid-phase extraction of phenolic compounds in water and milk samples. Reproduced from Ref. [104] with permission from *Journal of Molecular Liquids*. (**C**) Schema of procedure. Reproduced from Ref. [106] with permission from *Microchemical Journal*. (**D**) Schematic representation of the developed ferrofluid-based VALLME procedure. Reproduced from Ref. [108] with permission from *Talanta*. (**E**) Application in dispersive liquid–liquid microextraction of naphthalene-derived plant growth regulators in edible oil. Reproduced from Ref. [107] with permission from *Journal of Hazardous Materials*.

Research on liquid–liquid extraction focuses on optimizing novel solvents and improving dispersion efficiency, forming complementary systems for analytes with different polarities. Zheng et al. developed a self-dispersing DES ferrofluid (fenchol–hexanoic acid–acetic acid) that can rapidly disperse in water samples without vortex equipment. It extracts hexaconazole enantiomers through hydrophobic interactions (LOD = 0.006 μg/mL), addressing the reliance on dispersants in traditional liquid–liquid extraction [20]. These self-dispersing systems act similarly to sol–gel precursors, capable of solidifying target analytes within a localized matrix. Fan et al. constructed a superparamagnetic nanofluid using choline chloride/1-(o-tolyl)biguanide DES, which synchronously extracts short-chain and long-chain perfluoroalkyl substances (PFASs) in edible oils through weak N-H⋯F interactions, achieving recoveries of 90–109% and overcoming the selectivity contradiction caused by differences in PFAS chain lengths (Figure 8A) [100]. Alipanahpour et al. adopted a hydrophobic DES–ferrofluid system, enhancing doxycycline stability by adjusting pH to 3.0, enabling trace detection in urine and plasma (LOD = 3.6 ng/mL) [101]. Similarly, Alipanahpour et al. used a Fe_3_O_4_-OA-DES system to enrich mefenamic acid in urine through hydrogen bonding under pH 4.0 conditions (LOD = 1.351 ng/mL), covering both basic and acidic polar drugs [102].

Research on liquid–solid extraction focuses on the functional modification of magnetic particles to improve adsorption specificity and anti-matrix interference ability. For small-molecule pollutants in food and environmental matrices, Majidi et al. loaded alcohol-based DES onto SiO_2_@Fe_3_O_4_ to adsorb morin in fruit juices through hydrogen bonding (LOD = 0.91 μg/L) with an enrichment factor of 39.1 [103]. Yang et al. modified Fe_3_O_4_-OA with carbon quantum dots to enhance the π-π adsorption of phenolic compounds in water (LOD = 0.09–0.17 μg/L) (Figure 8B) [104]; the introduction of carbon quantum dots significantly improved particle dispersibility. For heavy metals and pesticides, Leal et al. used Fe_3_O_4_@graphene oxide combined with a DPTH chelating agent to simultaneously adsorb Cd^2+^/Pb^2+^ in high-salt seawater matrices (LOD = 0.005–0.008 μg/L) [105]. Wu et al. improved the enrichment factor of diazinon in fruit juices to 500 through chelation and π-π interactions of CLDH@Fe_3_O_4_@tannic acid, demonstrating the adaptability of composite modification to complex matrices (Figure 8C) [106].

In the future, integration with microfluidic chips could enable the on-line injection and separation of ferrofluids, while their self-dispersion property offers the potential for automation (Figure 8E) [107].

### 6.2. Friction Power Generation and Solar Thermal/Electro-Optical Conversion

With the advancement of renewable energy technologies, nanofluids and magnetofluids have emerged as key materials for enhancing energy conversion efficiency, thanks to their unique thermal, optical, and magnetic response properties [109,110,111]. Integrating these fluids into flexible gel matrices has paved the way for robust, wearable energy harvesters. Their core principles include: leveraging the triboelectrification effect at solid–liquid interfaces in triboelectric power generation (such as charge transfer between ferrofluids and polymers); in solar energy conversion, enhancing light absorption and thermal conduction through nanoparticles; and strengthening convective heat transfer via magnetic field regulation (e.g., modulation of Lorentz force and magnetic Rayleigh number) [14,68,112].

**Figure 9:**
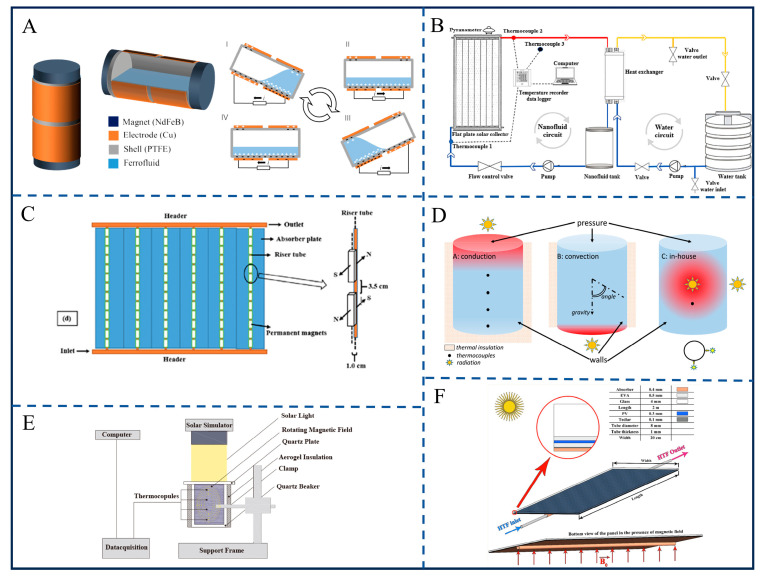
Schematic diagram of magnetic fluid friction power generation and solar thermal/electro-optical conversion functions. (**A**) Structure of the SF-TENG. Reproduced from Ref. [113] with permission from *Nano Energy*. (**B**) Schematic view of the experimental system. Reproduced from Ref. [114] with permission from *Energy Conversion and Management: X*. (**C**) A backside schematic of the manipulated collector without the presence of the insulation and the frame. Reproduced from Ref. [115] with permission from *Applied Thermal Engineering*. (**D**) Schematic presentation of heating alternatives and boundary conditions. Reproduced from Ref. [116] with permission from *Renewable Energy*. (**E**) Schematic of the system for evaluating the photothermal conversion performance of nanofluids. Reproduced from Ref. [117] with permission from *Energy Conversion and Management*. (**F**) Implement of magnetic field in PVT system. Reproduced from Ref. [118] with permission from *Journal of Magnetism and Magnetic Materials*.

Breakthroughs in magnetofluids within the field of triboelectric power generation have focused on ultra-low-frequency vibration energy harvesting. The solid–ferrofluid triboelectric nanogenerator (SF-TENG) proposed by Chen et al. utilizes triboelectrification between a polytetrafluoroethylene (PTFE) shell and ferrofluid (Figure 9A) [113]. By optimizing the ferrofluid volume (9 mL, accounting for 37.2% of the cavity) and magnetic field intensity (56 kA/m), it achieves an open-circuit voltage of 0.98 V and a power output of 1.03 nW under 0.5 Hz oscillation. Replacing the liquid ferrofluid in such devices with a soft, deformable ferrogel can prevent fluid leakage during continuous mechanical oscillation while maintaining the high surface contact area necessary for efficient triboelectrification.

In photothermal conversion, the synergistic effect of hybrid nanofluids is crucial: Ajeena et al. found that a ZrO_2_-SiC hybrid nanofluid in a flat-plate solar collector achieves a thermal efficiency of 75.21% at a concentration of 0.1% and a flow rate of 0.041 kg/s, which is 31.64% higher than that of distilled water (Figure 9B) [114]. When localized inside a thermo-responsive hydrogel, these photothermal nanoparticles can drive volume phase transitions, creating smart solar-actuated gel valves for advanced thermal management. For the Mn-Zn Fe_2_O_4_ ferrofluid studied by Shojaeizadeh et al. [115], under a 1.0 T magnetic field, the efficiency of the flat-plate solar collector is 52.15% higher than when using water (Figure 9C). Magnetic field regulation further enhances heat transfer: Balakin et al.’s CFD model shows that MnZn ferrite magnetofluid increases the efficiency of direct absorption solar collectors by 30% under an optimal magnetic field gradient [116], though an excessively strong magnetic field inhibits convection due to particle aggregation (Figure 9D); the α-Fe_2_O_3_ magnetic nano-rotor system designed by Wang et al. [117], driven by a rotating magnetic field, enables the binary nanofluid (RGO/α-Fe_2_O_3_/ethylene glycol) to achieve a photothermal efficiency of 56.8%, which is 14.5% higher than without a magnetic field, solving the problem of uneven temperature distribution in high-concentration nanofluids (Figure 9E).

In the field of photovoltaics, Jafaryar et al.’s numerical study demonstrates that the synergy between Fe_3_O_4_ nanofluid and magnetic fields can reduce photovoltaic (PV) module temperatures [118], increasing electrical efficiency by 0.78% and thermal efficiency by 6.31%. The increase in inlet velocity has the most significant impact on efficiency improvement (Figure 9F). Existing studies have confirmed that the “structural complementarity” of hybrid nanofluids and “active regulation” of magnetic fields are core to efficiency enhancement. Nanoparticle agglomeration causes performance degradation, which requires stability optimization through surface modification and dispersants—such as ultrasonic treatment in ZrO_2_-SiC fluids [114]. A critical matching value exists between magnetic field strength and concentration (e.g., 1.0 T with 0.8% concentration) [115]; excessive enhancement increases viscosity and pressure drop, necessitating the establishment of multi-parameter coupling models. In the future, exploring the application of ferrofluids in “vibration–photothermal” hybrid systems could enable synergistic energy harvesting across multiple scenarios via unified magnetic field regulation.

### 6.3. Optical Fiber Sensor

**Figure 10:**
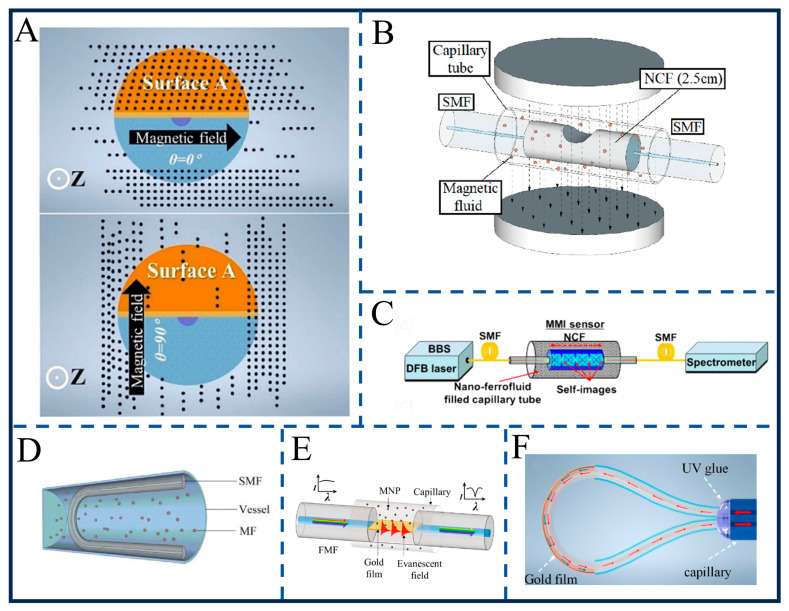
Schematic diagram of different magnetic fluid optical fiber sensors. (**A**) Schematic of MNP distribution around the sensor tip under 0° and 90° magnetic field. Reproduced from Ref. [129] with permission from *Measurement*. (**B**) Schematic diagram of the proposed sensing structure. Reproduced from Ref. [120] with permission from *Optics Express*. (**C**) Schematic of the MMI sensor setup for the measurement of transmitted spectrum. Reproduced from Ref. [132] with permission from *ACS Applied Nano Materials*. (**D**) Schematic diagram of U-bent SMF vector magnetometer. Reproduced from Ref. [133] with permission from *Optics Express*. (**E**) Schematic diagrams of the proposed sensor. Reproduced from Ref. [134] with permission from *Nanomaterials*. (**F**) Sensing structure of gold-clad bent multimode fiber. Reproduced from Ref. [135] with permission from *Materials*.

Magnetic field vector detection holds significant importance in fields such as industrial monitoring and geological exploration [119]. Traditional electromagnetic sensors are prone to interference, whereas fiber optic sensors have emerged as a research hotspot due to their advantages of anti-interference and high sensitivity [120,121,122,123]. The core principle lies in the directional aggregation of nanoparticles in magnetic fluid (MF) under the action of a magnetic field, leading to refractive index anisotropy [124,125,126,127]. Coating these optical fibers with a magnetic hydrogel layer allows for stable, long-term environmental monitoring without the risk of fluid evaporation or displacement. When combined with asymmetric fiber structures (e.g., bent or polished configurations), this phenomenon enables the conversion of magnetic field vector information into spectral shifts or intensity changes (Chen et al.) [128]. For instance, as shown in Figure 10A, the wedge-shaped fiber tip surface plasmon resonance (SPR) sensor designed by Wang et al. conducted a comparative study between multimode fiber (MMF) and few-mode fiber (FMF). It revealed that the anisotropic aggregation characteristics of magnetic nanoparticles (MNPs) in magnetic fluid—where MNPs are close to the fiber surface when the magnetic field is parallel to the polished surface and far from it when perpendicular—alter the SPR conditions [129]. This allows the FMF sensor to achieve a magnetic field intensity sensitivity of 6776 pm/mT and a directional sensitivity of 2313 pm/degree, significantly outperforming the MMF. Its high spatial resolution (approximately 657 μm) makes it particularly suitable for vector magnetic field detection in narrow spaces.

In recent years, researchers have developed multiple technical routes through structural innovation and material optimization. One category focuses on the anti-interference properties of polarization-maintaining fibers (PMF): Xu et al. proposed a single-end offset fusion-spliced structure of PMF and hollow-core fiber (HCF) [130]. When offset by 6 μm along the slow axis, the sensor achieves an intensity sensitivity of −813 pm/mT, a directional sensitivity of 245 pm/degree, and a temperature cross-sensitivity as low as 0.044 nm/°C, greatly enhancing its adaptability in industrial environments. Jiang et al. developed a long-period fiber grating (LPFG) based on thin-cladding polarization-maintaining fiber (TPMF) [131], regulating cladding mode resonance via magnetic fluid to achieve an intensity sensitivity of −618 pm/mT and a directional sensitivity of 72 pm/degree, with stable response enabled by the grating structure. Another category emphasizes simple structural design and high sensitivity: as shown in Figure 10C, Karki et al. designed a nano-ferrofluid-clad multimode interference (MMI) fiber optic sensor, with its fourth self-imaging spectral response optimized for the communication band. Leveraging the Néel and Brownian relaxation processes of the ferrofluid (with a response time < 1 ms), this sensor can detect AC magnetic fields up to 15 kHz, with a particular focus on the 60 Hz frequency of power grids. Under a 60 Hz magnetic field, it achieves a sensitivity of 240 mV/Gauss per dBm and an AC current sensitivity of 2.83 mV/A, enabling effective monitoring of current anomalies and faults in power grids [132]. Li et al. developed a U-bent single-mode fiber sensor [133], utilizing the interaction between whispering gallery mode (WGM) and magnetic fluid to achieve a directional sensitivity of 0.251 nm/degree and an intensity sensitivity of 0.517 nm/mT. This sensor requires no complex processing and exhibits excellent mechanical integrity (Figure 10D). As shown in Figure 10E, Chen et al. designed a side-polished few-mode fiber SPR sensor [134], enhancing response through functionalization with magnetic nanoparticles, achieving an intensity sensitivity of 0.692 nm/Oe and a directional sensitivity of −11.917 nm/degree. The SPR effect significantly improves detection capability for weak magnetic fields.

In addition, other studies have further enriched technical approaches. Liu et al. developed a gold-clad bent multimode fiber SPR sensor [135], achieving high-sensitivity detection by combining surface plasmon resonance (Figure 10F); Xiong et al. proposed a side-polished dual-core fiber interferometer, improving stability through mode interference [136]. In terms of dynamic response, Karki et al. explored the Néel/Brownian relaxation properties of magnetic fluids [137], providing a basis for broadband sensor design. Machine learning technologies have also been introduced, such as the use of random forest regression algorithms for defect depth reconstruction and k-nearest neighbor algorithms for magnetic field position classification [138], which significantly enhance detection accuracy. Vinod et al. systematically investigated the modulation mechanism of the magnetic field ramp rate on the thermal transport properties of oil-based ferrofluids, and found that a faster magnetic field ramp rate can induce the formation of high-density, fine-scale chain-like nanoaggregates [139]. A 230% enhancement in the thermal conductivity of the ferrofluid was achieved at a ramp rate of 33 G/s and a magnetic field strength of 200 G, and this thermal transport enhancement effect can be reversibly regulated via on–off cycling of the magnetic field, providing a brand-new technical route for the development of thermal control devices such as high-sensitivity heat flow sensors and intelligent cooling systems for high-power chips. For magnetic nanoemulsion systems, Mohapatra et al. revealed the generation mechanism of the magnetic field-induced optical transparency effect [140], confirmed that this effect originates from optical birefringence generated by the magnetic field-induced formation of rod-like anisotropic structures from nanodroplets, and clarified the power-law attenuation law between the critical magnetic field of the system and the volume fraction of nanodroplets, as well as the linear relationship between the normalized birefringence and the square of the applied magnetic field strength under a low magnetic field, laying an experimental and theoretical foundation for the design and development of low-power, fast-response optoelectronic devices including magneto-optic modulators, tunable optical filters and optical magnetic field sensors. Nandy et al. developed a thin-film sensing system based on magnetic nanoemulsions [141], which uses the stray magnetic field at the defects of ferromagnetic components to induce the oriented assembly of emulsion droplets into linear chain-like structures, and realizes rapid, non-contact, large-area visual detection of defects in carbon steel components through the Bragg reflection and visual color contrast generated by the structure. Meanwhile, they quantified the regulation law of emulsion stabilizing groups on the detection sensitivity and confirmed that the electrostatically stabilized system with 8 mM sodium dodecyl sulfate (SDS) exhibits the optimal defect detection sensitivity due to its shorter decay length of colloidal interaction force, with its defect width detection accuracy significantly superior to that of the traditional Hall probe-based magnetic flux leakage detection method, providing a new paradigm for the development of novel sensing systems for the non-destructive testing of ferromagnetic components, magnetic field visualization, and quantitative detection of trace magnetic analytes.

### 6.4. Heat Transfer Research

Ferrofluid exhibits regulable heat transfer performance under an external magnetic field, rendering it highly promising for applications in fields such as electronic cooling and heat exchangers [142,143,144,145,146,147,148,149,150]. The core principle lies in the ability of magnetic fields to induce the directional migration of nanoparticles, form chain-like aggregated structures, or disturb the flow field [151,152,153,154,155,156,157,158,159,160]. This chain-like aggregation is a form of magnetorheological gelation, where the fluid transiently transitions into a semi-solid gel state to establish highly conductive thermal pathways.

**Figure 11:**
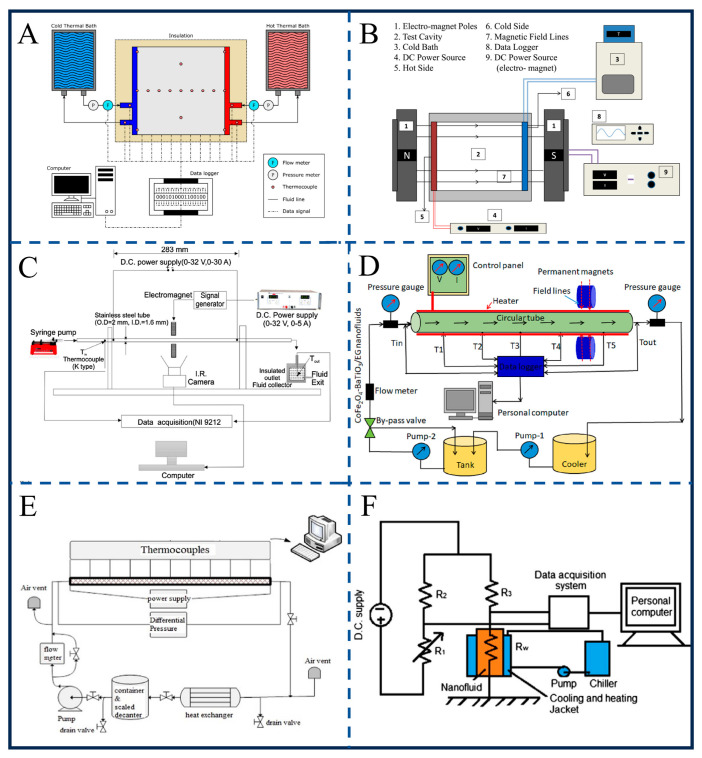
Schematic diagram of different heat transfer mechanisms of magnetic fluids. (**A**) Schematic view of the experimental setup. Reproduced from Ref. [161] with permission from *Journal of Magnetism and Magnetic Materials*. (**B**) Schematic of experimental setup. Reproduced from Ref. [162] with permission from *International Journal of Heat and Mass Transfer*. (**C**) Schematic of the experimental setup. Reproduced from Ref. [163] with permission from *International Journal of Heat and Mass Transfer*. (**D**) Schematic diagram of the experimental setup. Reproduced from Ref. [164] with permission from *Journal of Magnetism and Magnetic Materials*. (**E**) Schematic of experimental setup configuration. Reproduced from Ref. [166] with permission from *Journal of Magnetism and Magnetic Materials*. (**F**) Schematic diagram of the transient hot-wire method. Reproduced from Ref. [167] with permission from *Journal of Magnetism and Magnetic Materials*.

In terms of the regulation of heat transfer by magnetic field parameters, the regularities under different scenarios have gradually become clear. For constant magnetic fields, as shown in Figure 11A, Joubert et al. found in a differentially heated square cavity that the Nusselt number (Nu) of Fe_2_O_3_ nanofluid (0.1% volume fraction) is 5.63% higher than that of pure water without a magnetic field [161]. Integrating thermo-responsive gelators into these fluids could allow the system to automatically lock into a highly conductive solid gel state when critical temperatures are reached, preventing thermal runaway in electronics. Furthermore, a 700G permanent magnet in the “hot wall arranged above and below” configuration can further increase Nu by 2.81%. However, improper configurations (e.g., hot wall at the top and cold wall at the bottom) may hinder flow due to particle aggregation. As shown in Figure 11B, Dixit et al. further confirmed in a cubical cavity that the direction of the magnetic field significantly impacts its effect: when perpendicular to the hot and cold walls, it inhibits natural convection (Nu of 0.05% Fe_3_O_4_ decreases by 28%) [162]; when perpendicular to the direction of gravity, it enhances natural convection (Nu of 0.2% Fe_3_O_4_ increases by 28%). For alternating magnetic fields, Shyam et al. [163], using Fe_3_O_4_ nanofluid as the research object, found that heat transfer is enhanced by 39% at 0.1 Hz because the time scale of magnetic perturbation matches that of the flow (Figure 11C). In contrast, the effect at high frequencies (5 Hz) is comparable to that of a constant magnetic field.

In terms of fluid systems and synergistic effects, the advantages of hybrid ferrofluids and porous media are prominent. For the CoFe_2_O_4_-BaTiO_3_/ethylene glycol hybrid nanofluid studied by Sundar et al. [164], at a 1.0% concentration without a magnetic field, the Nusselt number (Nu) is 22.19% higher than that of the base fluid (Figure 11D). When a 4000G magnetic field is applied near the inlet (*x*/*d* = 30), the total increase in Nu reaches 72.33%, while the friction factor rises to 68.75%—confirming the “magnetic–high thermal conductivity” synergistic mechanism of hybrid particles [165]. As shown in Figure 11E, Sheikhnejad et al. found that the synergy between copper foam porous media and a magnetic field in a horizontal tube can increase the heat transfer coefficient of Fe_2_O_3_ nanofluid by 2.4 times (compared to 2.2 times with porous media alone and 1.4 times with magnetic field alone) [166]. In terms of thermophysical properties, Sundar et al. measured that the thermal conductivity of Fe_3_O_4_/water at a 2.0% concentration and 60 °C is 48% higher than that of pure water, while its viscosity is 2.96 times that of pure water (Figure 11F) [167].

Current research has elucidated the coupling mechanism of magnetic field–particle-flow field, yet there remains room for further exploration. Although the synergistic effect of hybrid ferrofluids (e.g., CoFe_2_O_4_-BaTiO_3_) has been confirmed, the dynamic matching rules between their particle ratios and magnetic field parameters require in-depth investigation. Additionally, the issue of magnetic attenuation in high-temperature scenarios needs to be addressed through particle coating technology [168], laying the groundwork for the large-scale application of ferrofluids in high-power equipment.

### 6.5. Magnetic Seal

Ferrofluid is composed of nanoscale magnetic particles, a base carrier liquid, and a surfactant, embodying both liquid fluidity and magnetic properties. Under the influence of a magnetic field, it can be confined within sealing gaps to form a liquid film, achieving non-contact sealing by resisting pressure differentials across both sides [169]. Owing to characteristics such as zero leakage and long service life, it is widely applied in fields like aerospace and machinery. Its sealing performance depends on the magnetic field’s ability to constrain the ferrofluid [21,170]. However, in practical applications, influenced by centrifugal force, structural parameters, and environmental factors, issues such as liquid film rupture and insufficient pressure resistance are prone to occur. Thus, in-depth research into failure mechanisms, structural optimization, and material properties is necessary.

**Figure 12:**
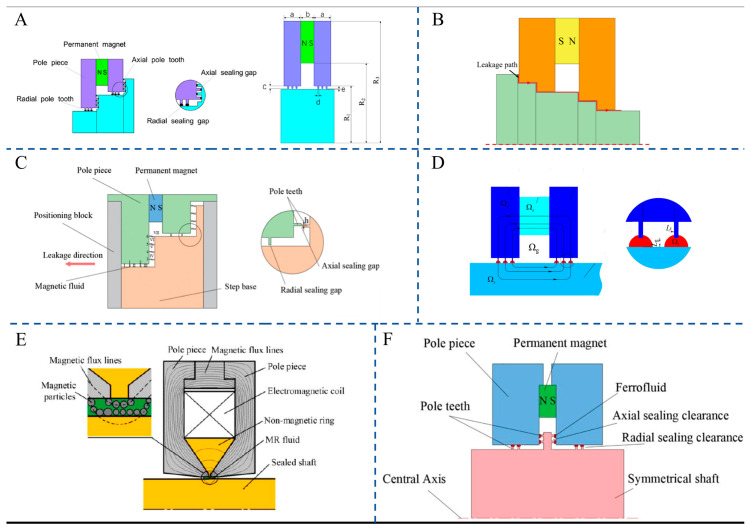
Diagram of different magnetic sealing devices. (**A**) Interlaced FS structure and common FS structure. Reproduced from Ref. [171] with permission from *Vacuum*. (**B**) Schematic diagram of the leakage path. Reproduced from Ref. [173] with permission from *Tribology International*. (**C**) Structure of CFFS-SPT. Reproduced from Ref. [174] with permission from *Tribology International*. (**D**) Structure diagram of the ferrofluid seals. Reproduced from Ref. [175] with permission from *Tribology International*. (**E**) The schematic illustration of sealing structure. Reproduced from Ref. [176] with permission from *Journal of Magnetism and Magnetic Materials*. (**F**) Principle of SFFS structure. Reproduced from Ref. [177] with permission from *Tribology International*.

Existing studies have significantly enhanced sealing performance through structural innovation. As shown in Figure 12A, Yang et al. designed an interlaced ferrofluid seal that utilizes a labyrinth-like leakage channel to reduce ferrofluid loss [171]. Its self-repairing performance is superior to that of conventional structures: when equipped with five axial pole teeth, three radial pole teeth, and a 0.2 mm gap, it can still withstand pressure exceeding 1 atm after the third repair, making it suitable for vacuum environments. This grease effectively functions as a thixotropic gel, demonstrating how gel-like rheological properties natively enhance dynamic sealing capacity. Similarly, in the convergent structure with alternating pole teeth, stepped shaft end teeth and axial pole teeth form a synergistic constraint. The saturation value of the ferrofluid injection volume is 1.5 mL, and the critical pressure increases with the number of pole teeth, reaching 300 kPa—1.71 times that of ordinary stepped seals [172]. The stepped pole piece design achieves a maximum critical pressure of 180 kPa (9.98 times that of ordinary seals) by extending the leakage path, with the optimal self-repairing ratio (0.94) observed when there are three steps and a 0.2 mm axial gap (Figure 12B) [173]. Liu et al. further revealed through visualization experiments that, in the interlaced pole tooth structure [174], pressure transfer in the radial chamber is faster than in the axial chamber, and failure occurs in two stages: “micro-leakage” and “complete leakage”. Increasing the eccentricity distance of axial pole teeth can enhance critical pressure (reaching 123.67 kPa at 0.6 mm), while adding magnetic isolation materials to the tooth grooves suppresses magnetic leakage, further improving pressure resistance (Figure 12C).

Research on failure mechanisms and material expansion has enriched sealing theory. As shown in Figure 12D, Cheng et al. established a bidirectional coupled multi-physics model [175], revealing that centrifugal force causes ferrofluid to detach from the shaft surface along the pole teeth, with the highest radial velocity occurring at the liquid film boundary. They proposed dimensionless numbers to quantify the relationship between centrifugal force, pressure, and magnetic field force, reducing calculation errors to 3.29–13.5% compared with traditional methods. Liu et al. also found that pressure transfers stepwise along the axial chamber and rises almost synchronously in the radial chamber, differing from the traditional sealing mechanism. The self-repairing capability decreases with the number of ruptures due to ferrofluid loss (Figure 12E) [21]. In terms of materials, the micro–nano composite magnetorheological grease prepared by Wang et al. exhibits a viscosity increase of more than 10 times under a magnetic field [176]. Increasing the sealing gap (from 0.1 mm to 1.0 mm) reduces pressure resistance from 70 kPa to 20 kPa. Enlarging the pole tooth width can extend low-pressure stability, but the axial magnetic field component weakens actual sealing performance. Furthermore, in multi-tooth structures, pressure transfers chamber by chamber, and the fluid barrier undergoes a “rupture–recovery” cycle (Zhou et al.), consistent with the aforementioned description of failure stages (Figure 12F) [177].

Cutting-edge research indicates that the core of ferrofluid sealing lies in the synergistic regulation of “magnetic field confinement–structural design–material properties”. Although current structural optimization has significantly improved pressure resistance and self-repairing performance, the multi-field coupling mechanism under dynamic working conditions remains unclear. For example, the impact of temperature and vibration on liquid film stability has not yet been quantified. Magnetorheological grease, despite its advantage in anti-sedimentation performance, has not achieved breakthroughs in integrated applications with structures such as interlaced pole teeth. In the future, combining AI algorithms could enable the global optimization of pole tooth parameters and gap sizes, while exploring the synergistic design of material–structure–magnetic field under extreme environments (low temperature, high pressure) will promote the leap from laboratory research to engineering applications.

### 6.6. Other Applications

**Figure 13:**
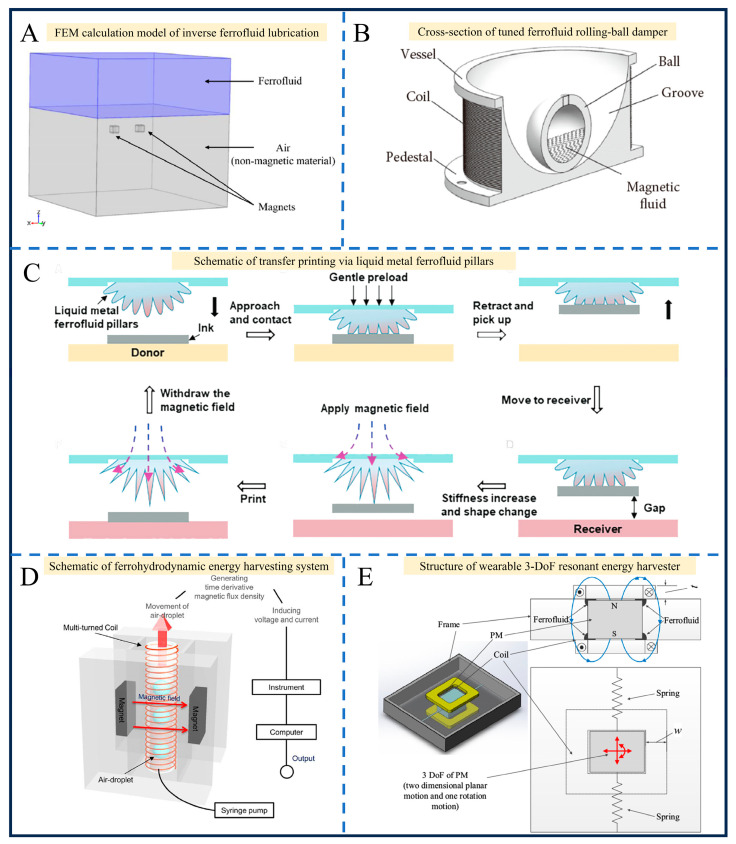
(**A**) Computational box where the magnetic field is obtained using the Finite Element Method. The ferrofluid (blue region) is placed exactly 2 mm above the pair of magnets. The distance of 2 mm corresponds to the thickness of the steel plate. Reproduced from Ref. [186] with permission from Tribology International. (**B**) The 3D structure section of the tuned magnetic fluid rolling-ball damper. Reproduced from Ref. [187] with permission from Advances in Materials Science and Engineering. (**C**) Schematic illustration of magnetically actuated noncontact transfer printing strategy enabled by the liquid metal ferrofluid pillars. Reproduced from Ref. [188] with permission from Advanced Materials. (**D**) A schematic illustration and working principle of ferrohydrodynamics based energy harvesting system. Reproduced from Ref. [189] with permission from Nano Energy. (**E**) Structure of proposed harvester. Reproduced from Ref. [190] with permission from Applied Energy.

In addition, ferrofluids exhibit significant application potential in fields such as lubrication, vibration control, energy harvesting, and precision manufacturing [178,179,180]. In the field of non-destructive testing (NDT), the core application of ferrofluids is the magnetic flux leakage (MFL) detection of ferromagnetic materials [181]. Its core working principle is that, when defects such as cracks and corrosion occur in ferromagnetic components, a magnetic leakage field will form at the defect sites. The magnetic nanoparticles in the ferrofluid directionally migrate and aggregate towards the defects under the action of the magnetic leakage field, thereby achieving high-sensitivity visual detection of micro-defects [182,183]. Compared with the conventional dry magnetic particle testing technology, ferrofluid-based detection technology has the advantages of higher defect resolution, stronger adaptability to components with complex curved surfaces, and faster detection speed [184]. At present, this technology has been industrially applied and widely used for defect detection and safety assessment in critical facilities including oil and gas transmission pipelines, railroad tracks, pressure vessels, and metal storage tanks [141]. In the field of lubrication, the magnetic response characteristics of ferrofluids are widely utilized to optimize tribological properties. Huang Xingbao et al. established an oil-based ferrofluid lubrication model for spur gear drives and found that, compared with pure base oil [185], ferrofluid can significantly increase film thickness and normal stiffness. Furthermore, ferromagnetic particles with small sizes (10 nm) and high bulk concentrations (10%) effectively reduce the friction coefficient and enhance anti-wear capability. Although increasing the magnetic field intensity has little impact on film thickness, it can further optimize lubrication effects through the oriented arrangement of particles. As shown in Figure 13A, Sahoo et al. proposed an inverse ferrofluid design, in which non-magnetic lubricating particles (e.g., PTFE, MoS_2_) are dispersed in ferrofluids [186]. By leveraging magnetic field gradients, these particles aggregate in the contact area, enabling local regulation of the friction coefficient. Under the boundary lubrication regime, the wear rate is reduced by over 20%, providing a novel approach for precision lubrication under extreme working conditions. Both studies indicate that ferrofluid lubrication performance can be synergistically regulated through particle parameters and magnetic fields, albeit via distinct mechanisms: the former relies on the lubrication-enhancing effect of ferromagnetic particles themselves, while the latter achieves local reinforcement through the magnetically controlled aggregation of non-magnetic particles.

The tuned magnetic fluid rolling-ball damper developed by Yang et al. realizes vibration control through a magnetic fluid–solid coupling model (Figure 13B) [187]. Magnetic hydrogels are highly suited for this type of vibration control, as their tunable viscoelasticity allows them to absorb shock efficiently without relying on a rigid container. Jiang et al. fabricated liquid metal ferrofluids into columnar adhesive materials [188], utilizing magnetic field-induced abrupt stiffness changes (from 0.025 N/mm to 1.299 N/mm) to achieve non-contact transfer printing (Figure 13C). This process explicitly mimics a phase transition, stiffening the fluid into a temporary gel pillar to gently pick up fragile objects, further proving the indispensability of gelation mechanics in advanced manufacturing and soft robotics. As shown in Figure 13D, Hye Rim Yun et al. proposed an energy harvesting system based on ferrohydrodynamics and magnetorheological effects [189], which generates electrical energy by disturbing the magnetic field through the movement of air droplets in ferrofluids, following Faraday’s law of electromagnetic induction. They analyzed the influence of four forces (buoyancy, interfacial tension, drag, and yield force) on droplet motion, comparing two ferrofluids (EFH-1 and EFH-3) and finding that EFH-3 performed better due to its higher saturation magnetization and particle concentration. Magnetic field intensity affects output amplitude, while flow rate influences frequency, with conversion efficiency increasing with coil turns—making it suitable for scenarios like wind tunnels. Meanwhile, Shuai Wu et al. designed a wearable three-degree-of-freedom (3-DoF) electromagnetic resonance energy harvester for human motion, which uses ferrofluids to achieve “contactless” suspension of permanent magnets (PM) to reduce friction (withstanding 10 times gravitational acceleration) (Figure 13E) [190]. Its three DoFs match multi-frequency human movements, with ferrofluids improving harvesting efficiency by 40% (walking) and 20% (running), generating an average power of 2.28 mW when running, and the energy storage circuit supports wearable sensors. Both studies rely on the magnetic response characteristics of ferrofluids: the former focuses on energy conversion via droplet movement in fluids, while the latter targets wearable scenarios for human motion, with the magnetorheological effect and low-friction properties of ferrofluids being key to efficiency improvement.

Current research has confirmed the feasibility of ferrofluids in multiple fields. In the future, through interdisciplinary integration, ferrofluids are expected to facilitate more extensive applications in fields such as intelligent equipment and biomedicine.

## 7. Conclusions and Outlook

This article focuses on magnetic nanomaterials, ferrofluids, and their evolution into ferrogels and magnetic hydrogel systems, systematically exploring their structural characteristics, dynamic behaviors, preparation techniques, and applications across medical and engineering fields. Structural characterization via electron microscopy (SEM, TEM) reveals that magnetic nanomaterials typically exhibit nanoscale spherical features, with particle size (4–198 nm) and dispersibility closely linked to synthesis methods. For instance, thermal decomposition and solvothermal methods yield particles with uniform size distributions, while surface modifications such as triethylene glycol coating enhance monodispersity—factors that directly influence functional efficiency, including hyperthermia specific absorption rate (SAR) values. For ferrofluids and their gel counterparts, macroscopic behaviors (e.g., deformation, spike formation, sol–gel phase transitions) and microscopic mechanisms (e.g., dipolar chain formation, interfacial tension changes) are regulated by parameters including the Weber number, magnetic Bond number, and Ohnesorge number. Magnetic fields induce shape instabilities, droplet wobbling, and migration, with numerical simulation methods effectively capturing dynamic processes such as coalescence.

Ferrofluid preparation methods fall into two categories: green synthesis (utilizing natural iron sand, emphasizing low cost and eco-friendliness) and chemical reagent synthesis (enabling precise control over particle size, composition, and sol–gel network formation). Surface modification strategies (e.g., double-layer coating, ligand exchange) improve stability and biocompatibility to support functional applications, serving as ideal precursors for robust hydrogel networks. Applications span both medical and engineering fields: in medicine, these include targeted hyperthermia, pH-responsive magnetic gel drug delivery (nearly 100% release at pH 5.0), and T1-T2 dual-mode MRI; in engineering, they encompass efficient extraction, triboelectric power generation, high-sensitivity fiber optic sensing, and magnetically regulated heat transfer.

Despite such progress, several key challenges persist. Material stability and biocompatibility remain critical issues in fluid systems; however, encapsulating these nanoparticles within three-dimensional gel matrices significantly mitigates agglomeration in complex environments (e.g., biological fluids or high-salt matrices) and prevents nanoparticle leaching. Additionally, long-term nanomaterial toxicity and poor stability in large-scale production hinder clinical translation and engineering applications—for example, NdFeB particles require SiO_2_ coating to improve biocompatibility, and ferrofluid stability in biological media demands further enhancement. The correlation between microstructures and macroscopic behaviors is insufficiently understood: real-time links between microscopic processes and macroscopic phenomena under dynamic magnetic fields remain unclear, while multi-field coupling mechanisms in heat transfer and energy conversion lack systematic theoretical models. Technical limitations in preparation and characterization also persist, as existing methods struggle to balance precision and scalability.

To address these challenges, priority should be given to developing multifunctional, multi-stimuli-responsive magnetic gel materials. Designing systems that integrate magnetic responsiveness with sensitivity to pH, temperature, or light will enhance targeting and environmental adaptability—for example, combining thermosensitive liposomes with thermo-responsive ferrogels to achieve the closed-loop regulation of “temperature-triggered release, magnetothermal enhancement, and imaging monitoring”. Crucially, a highly promising future direction is research on the drive–perception–control collaborative mechanism of multi-energy field-responsive microrobots. Utilizing soft ferrogels to create these microrobots will enable precise drug delivery through complex physiological barriers. Strengthening mechanistic research and model construction is also vital: establishing multi-field coupling numerical models to clarify micro–macro correlations in ferrofluids and magnetorheological gels under dynamic magnetic fields, and developing in situ characterization technologies to capture real-time structural changes in complex environments will provide a solid theoretical basis for precise regulation.

## Figures and Tables

**Table 1:** Comparison of key magnetic parameters from VSM characterization for typical magnetic nanoparticles and their corresponding ferrogels.

Material System	Saturation Magnetization *M_s_* (emu/g)	Corresponding Reference
Fe_3_O_4_	54	[43]
Fe_3_O_4_@TSTC[4]AS-s-SA sodium alginate-based superparamagnetic nanocomposite	45.6	[43]
Copper ferrite (CuFe_2_O_4_) nanoparticles	80	[45]
Uncoated (bare) cobalt ferrite (CoFe_2_O_4_) nanoparticles	44	[44]
Folic acid-chitosan-coated CoFe_2_O_4_ nanoparticles	40	[44]
Fe_3_O_4_-filled polyacrylamide ferrogel	2.18~4.37	[46]

## Data Availability

No new data were created or analyzed in this study. Data sharing is not applicable to this article.
